# Dual Posteromedial Portal Technique: Arthroscopic Loop Suspensory Fixation–Posterior Cruciate Ligament Tibial Avulsion Fracture

**DOI:** 10.1016/j.eats.2024.103187

**Published:** 2024-08-19

**Authors:** Nuno Ramos-Marques, Vicente Campos, Thiago Aguiar, João Torrinha Jorge

**Affiliations:** a Department of Orthopedic Surgery, Hospital de Curry Cabral, Unidade Local de Saúde São José, Lisboa, Portugal; b Department of Orthopedic Surgery, Hospital da Luz Torres de Lisboa, Lisboa, Portugal

## Abstract

Posterior cruciate ligament avulsion fracture injuries have been associated with an increased risk of meniscal tears and premature patellofemoral/medial compartment osteoarthritis. Sports-related trauma is a common cause of posterior cruciate ligament avulsion fractures. Surgical management is recommended for displaced bony avulsion of the posterior cruciate ligament to stabilize the knee and prevent knee pain, nonunion, and osteoarthritis progression. This article discusses our preferred treatment using a loop suspensory fixation device through a dual posteromedial portal technique.

A posterior cruciate ligament (PCL) avulsion fracture, although rare, is a debilitating condition that, if not treated, will lead to instability and early degenerative changes.^1^ The PCL is responsible for limiting posterior tibial translation, leading to rotation stability and posterior stability during motion at all knee position planes. Tibial PCL avulsion fracture is the most common form of isolated PCL bony avulsion, and concomitant intra-articular knee injuries are frequently associated, such as meniscal tears or loose bodies.

As such, early surgical treatment of displaced PCL avulsion fracture is necessary to restore biomechanics, prevent early degenerative changes, provide knee stability, and ensure adequate bone healing.

There are multiple surgical techniques available, depending on the size, comminution, amount of displacement of the fracture fragment, and concomitant intra-articular injuries.

Although conventional open surgical fixation of PCL avulsion fracture has been described with satisfactory clinical outcomes, due to the proximity of the popliteal neurovascular anatomic structures, these carry a significant surgical risk. Arthroscopic surgical fixation of isolated PCL avulsion fractures seems to mitigate the risks associated with open surgical approaches with similar or even better outcomes since there is also the possibility to address intra-articular injuries.

To visualize and confirm an accurate fragment reduction/fixation, a posteromedial portal is obligatory. The most important structure in posterior compartment arthroscopic surgery is the popliteal neurovascular anatomic structure. Of these structures, the popliteal artery is the most anterior structure of the popliteal neurovascular bundle and the most susceptible to harm while the surgeon is working.

There is also the possibility to utilize a posterolateral portal through the posterior septum by pushing through the loose areolar tissue behind the PCL. The posterior septal fibrous tissue, however, is thick and resistant in some cases, which greatly impedes the passage and increases the risk of capsular and/or popliteal neurovascular injury.^2^

To avoid this catastrophic complication, the portals should be established with the knee flexed to 90°.^3^

Regarding fixation devices, such as cannulated screws, anchors, sutures, and suspensory fixation methods, the cortical suspension button and retrograde screw fixation techniques have shown comparable structural properties to the direct screw fixation technique.

Therefore, we describe our preferred treatment using a loop suspensory fixation device through a dual posteromedial portal technique.

## Surgical Technique

### Patient Evaluation

An examination under anesthesia is performed on the operative knee, including examination for posterior laxity as well as assessing for varus/valgus stability at 0° and 30°. The addition of rotation to the posterior drawer is used as a further test to assess the integrity of the PCL and posterior corners of the knee.

### Imaging

**Fig 1:**
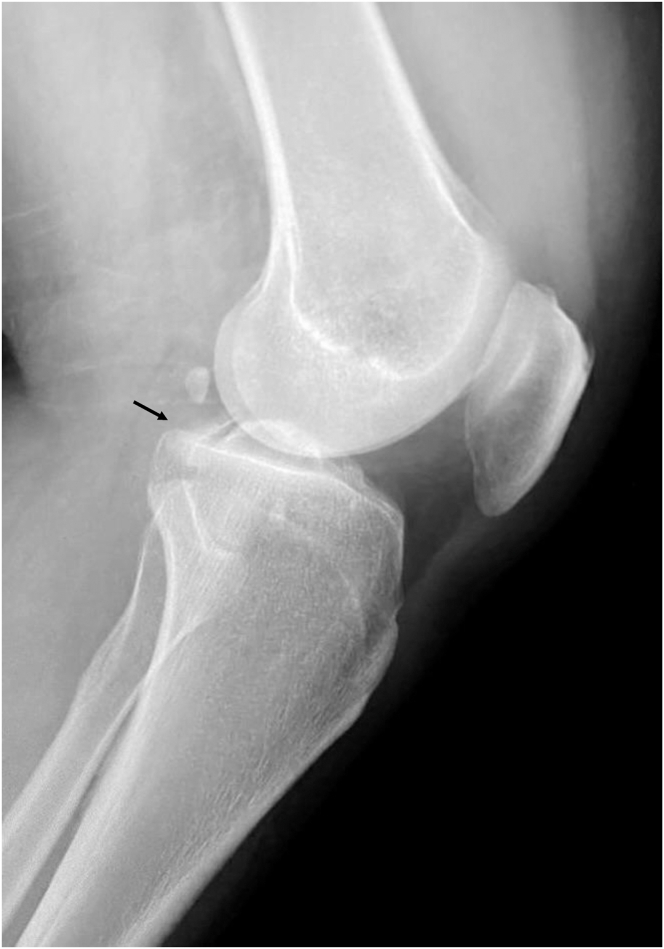
Lateral view radiograph of the right knee, showing a displaced posterior cruciate ligament tibial avulsion fracture injury. In the presence of minimally displaced fractures, fluoroscopic stress views can be obtained to evaluate for posterior cruciate ligament laxity.

**Fig 2:**
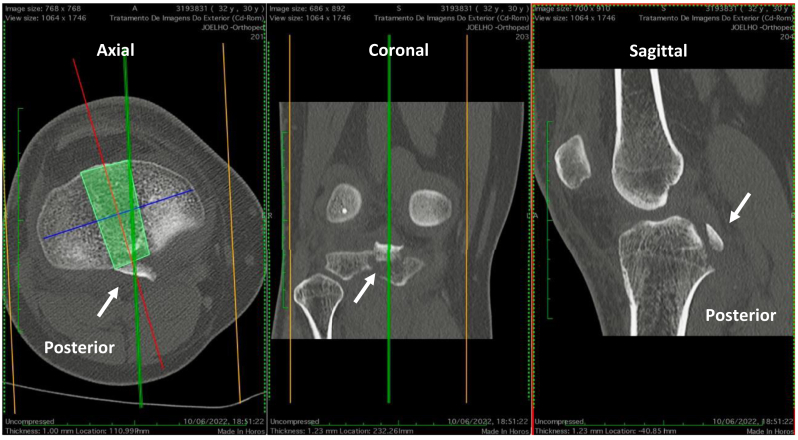
Computed tomography scan demonstrating the posterior cruciate ligament tibial avulsion fracture, including its size, grade of comminution, and the amount of displacement, an integral part of the preoperative imaging studies to plan the surgery.

A standard knee series, including anteroposterior, Rosenberg, lateral (Fig 1), and Merchant patellar radiographs, should be evaluated for any evidence of avulsion fractures and associated knee injuries, but computed tomography scans continue to play a major role in the assessment of PCL injuries with associated fractures or avulsions (Fig 2).

The information acquired from magnetic resonance imaging scans is invaluable for preoperative planning, specifically to identify associated tendon, ligament, meniscal, and cartilage injuries, as well as the mechanism of injury, particularly the location of bone bruising patterns.

### Indications

Patients were included if they had a PCL tibial avulsion fracture, were physically active, were within 2 weeks of injury, and did not have grade III/IV osteoarthritis according to the Kellgren-Lawrence classification.

### Surgical Description

**Fig 3:**
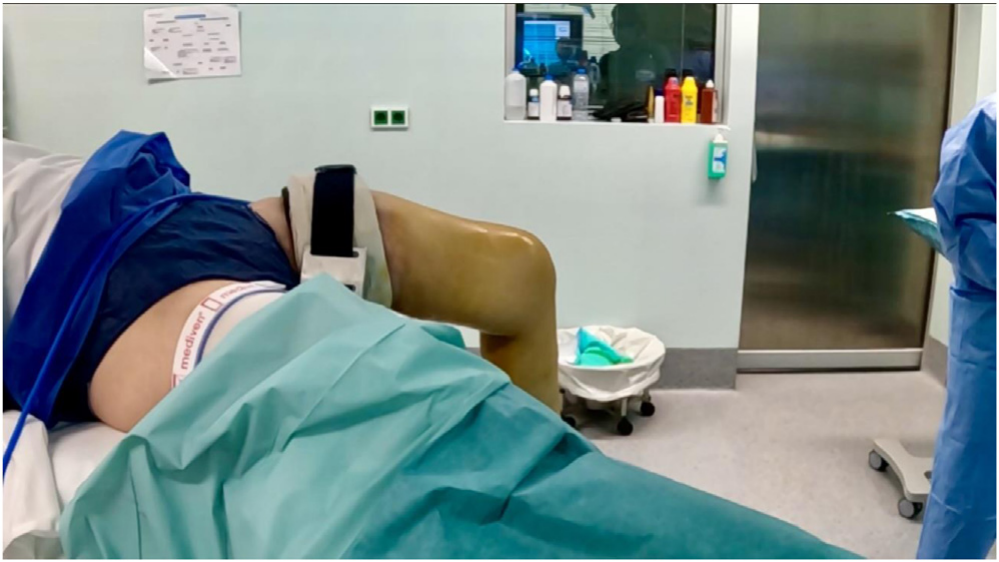
A well-padded high thigh tourniquet is placed. A thigh holder and unobstructed motion up to 120° is checked. The other thigh is abducted and supported after adequate padding. This position allows for an unobstructed working area for the surgeon, especially with the dual posteromedial portal technique, and for the assistant when it comes to the reduction phase, allowing a proper anterior tibial translation while keeping the knee in 90° of flexion.

The patient is brought into the operating room, placed in the supine position, and induced under general anesthesia. Cefazolin (2 g) is given before the incision. A well-padded high thigh tourniquet is placed. Range of motion is assessed under general anesthesia, as dictated in the patient evaluation subsection. A thigh holder and unobstructed motion up to 120° is checked. The other thigh is abducted and supported with a leg holder after adequate padding (Fig 3). The tourniquet is inflated throughout the procedure. The surgical technique is demonstrated in Video 1.

After standard anterolateral and anteromedial portals are established, a thorough intra-articular knee examination is done to assess possible concomitant intra-articular injuries. Subsequently, the 30° arthroscope can be advanced in the posteromedial compartment through the Gillquist portal or modified Gillquist maneuver or using the window through the anterior and posterior cruciate ligament–intercruciate interval.

The double posteromedial portal^4^^,^^5^ is established while examining the posterior compartment through the Gillquist portal.

The fracture is exposed by releasing some of the soft tissue remnants, fracture debris, and blood clots using an arthroscopic radiofrequency wand.

**Fig 4:**
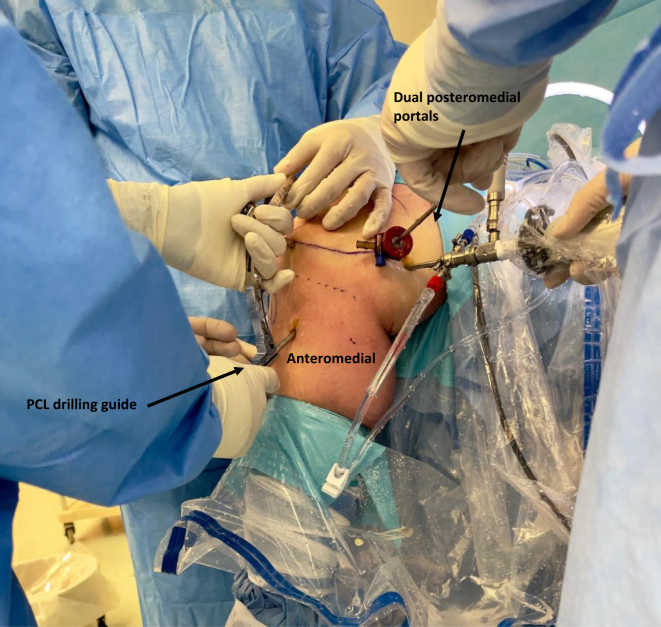
An arthroscopic posterior cruciate ligament tibial drill guide is passed through the anteromedial portal and used to reduce the fracture. The reduced fracture fragment is then pinned in position using the accompanying 2.4-mm guide pin under direct arthroscopic visualization via the posteromedial portal. (PCL, posterior cruciate ligament.)

An arthroscopic PCL tibial drill guide (ACUFEX; Smith & Nephew) is passed through the anteromedial portal and used to reduce the fracture (Fig 4). The reduced fracture fragment is then pinned in position using the accompanying 2.4-mm guide pin under direct arthroscopic visualization via the posteromedial portal. This is then over-reamed with a 4.5-mm reamer. The reamer is then left in situ and the guide pin is removed. A wire loop is then introduced through the reamer into the posterior compartment and retrieved from the posteromedial portal. A suspensory device is then relayed through the tibia tunnel with a suture shuttle. The oblong button (Xtendobutton; Smith & Nephew) is then flipped onto the bony fragment under direct arthroscopic visualization. A button on the anteromedial tibia is then tensioned by sequentially pulling on both limbs of the device to fix the fracture in its reduced position. The knee is cycled between flexion and extension multiples times to assess the tension given through the suspensory device under direct arthroscopic visualization. The tensioning sutures are then tied with a Nice knot before completing multiple half hitches to secure the fixation.

**Fig 5:**
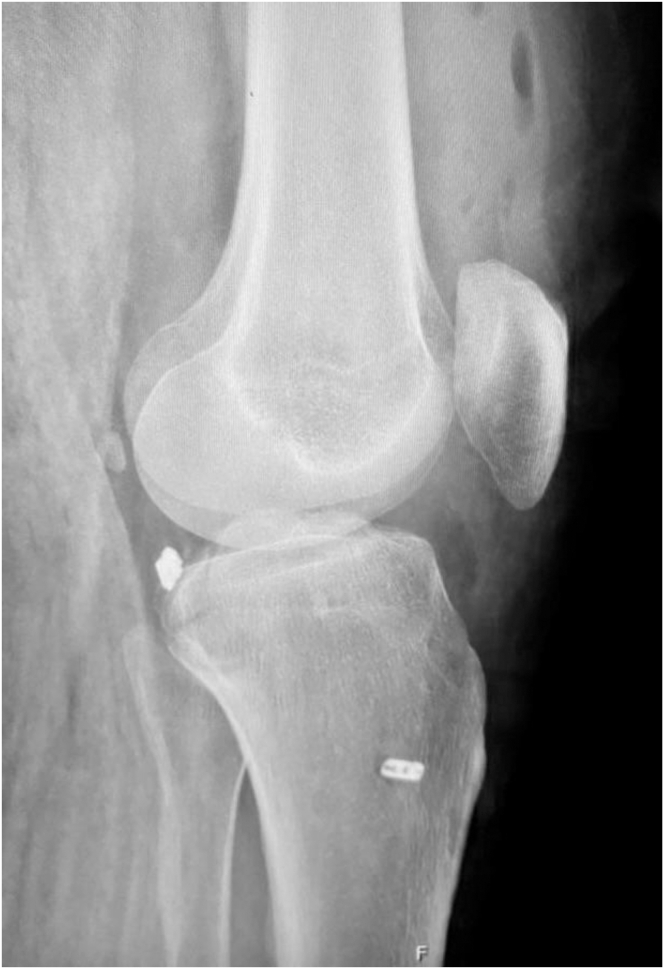
Final fluoroscopic images, such as a lateral view, are obtained to confirm the reduction and correct trajectory and position of the final suspensory device. While fluoroscopic images are not mandatory, they allow us to have a broader view of our surgery’s end result.

Although not necessary, final fluoroscopic images are obtained to confirm reduction and final suspensory device position (Fig 5).

The deep tissues are closed with 0 and 2-0 Vicryl (Ethicon), followed by staples for the skin. A sterile dressing is applied.

The knee joint is fixed with a dynamic PCL brace after the operation, and isometric contraction of the quadriceps femoris and straight-leg raising exercises are encouraged as soon as possible. Hyperextension should be avoided (12 weeks). Isolated hamstring exercises should be avoided until week 12 of weightbearing. Partial weightbearing can be done with crutches (2 weeks). Prone passive range of motion from 0° to 90° is allowed for the first 2 weeks, with progression to full range of motion. A dynamic PCL brace is to be worn at all times, including rehabilitation and sleep (minimum of 12 weeks). After 12 weeks, the knee joint can be moved freely, and strength exercises are performed at the same time.

Deep vein thrombosis prophylaxis with enoxaparin for 5 weeks is performed. Anteroposterior and lateral radiographs are obtained on postoperative day 1, week 2, and week 8 (Fig 5).

## Discussion

A systemic review found that either surgical approach (open vs arthroscopic) renders similar outcomes and risks for patients with displaced tibial-sided PCL avulsion fractures. The arthroscopic approach group did have a higher-grade A, or normal knee, outcome score on the International Knee Documentation Committee (IKDC) when compared with the open treatment group (78.9% vs 65.9%), but patients undergoing arthroscopic-only treatments may also experience higher rates of arthrofibrosis. The clear advantage of the arthroscopic approach is that concomitant intra-articular injuries may be addressed at the time of the index operation.^6^

A case series study by Zhu et al.^7^ with 30 patients showed that the treatment of PCL avulsion fractures with arthroscopic adjustable-loop cortical button fixation is easy to perform and showed good clinical results: Lysholm knee function score was 45.93 ± 6.15 before surgery and 87.10 ± 3.71 at 12 months after surgery, and the IKDC score was 19.27 ± 4.40 before surgery and 95.47 ± 1.87 at 12 months after surgery, with a statistically significant difference.

Another case series study by Han et al.^8^ showed that all fractures achieved union. Mean postoperative Lysholm score was 91.5 (range, 85-95), IKDC score was 85.1 (range, 74.7-89.7), and Knee injury and Osteoarthritis Outcome Score was 89.3 (range, 81.5-94.6). All patients returned to their preinjury activities of daily living and work.

Fracture fragmentation is a possible and frightening problem during the drilling part of the surgery. Therefore, other types of fixation are sometimes necessary to reduce the PCL avulsion fracture, but if the fragments are contained by the PCL, we believe that augmenting the button with an oversized button, such as G-lok-XL (Stryker) or Xtendobutton (Smith & Nephew), is a pleasant solution, and we have not found any published solution regarding this problem (Table 1). Besides, a larger surface area due to the longer length and width of the button allows even compression along wider fracture beds.

**Table 1:** Pearls and Pitfalls

Pearls	Pitfalls
Place the ACL/PCL guide through the anteromedial portal and reduce the avulsion fracture through direct arthroscopic visualization.	Incision placed too low or far from the midline will lead to a more angulated entry through the posterior compartment and the view might be insufficient.
Two posteromedial portals make the popliteal neurovascular bundle injury less likely, allowing for proper visualization and clearance of the PCL footprint while avoiding additional posterolateral portal creation.	Avoid being too inferior in PM portal creation. Saphenous nerve or its sartorial branch injury is a possibility.
Button augmentation allows the treatment of different fracture patterns (larger or comminuted) and distributes the tension throughout a wider surface area.	Dual PM portals will have slightly higher risk of saphenous nerve injury compared with a single portal technique, but following the safe zone technique with respect to synovial folds completely minimizes the saphenous nerve injury.
Intercruciate interval window is necessary to properly pass the PCL guide and reduce the fracture.	The proximal portal may need a long cannula in patients with a high body mass index.

ACL, anterior cruciate ligament; PCL, posterior cruciate ligament; PM, posteromedial.

The report of Ezechieli et al.^9^ confirmed that, compared with the suture group, the initial displacement of the fixation group was significantly lower with a greater maximum failure load and better effect than that of the suture group.

Besides the popliteal neurovascular bundle injury risk during posterior compartment arthroscopic surgery, a higher arthrofibrosis risk is also reported when compared with open approaches. The most common complication in both groups was arthrofibrosis, which was reported more often in the arthroscopic group (0%-35%) than the open treatment group (0%-25%).^6^

Overall, arthroscopic-assisted PCL avulsion fracture fixation with a suspensory device is a reproducible surgical treatment option, with patients able to expect normal or near-normal knee outcome scores, complete bone healing, and knee stability restoration (Table 2).

**Table 2:** Advantages and Disadvantages

Advantages	Disadvantages
Fluoroscopy imaging is unnecessary with arthroscopic-assisted reduction.	Higher risk of arthrofibrosis.
Suspensory devices show comparable fixation strength to antegrade screws.	Risk of injury to popliteal neurovascular structures.
Early mobilization when compared with open procedures.	Technically challenging procedure.

## Disclosures

All authors (N.R-M., V.C., T.A., J.T.J.) declare that they have no known competing financial interests or personal relationships that could have appeared to influence the work reported in this paper.

## Supplementary Data

Video 1 A well-padded high thigh tourniquet is placed. A thigh holder and unobstructed motion up to 120° is checked. The other thigh is abducted and supported after adequate padding. After standard anterolateral and anteromedial portals are established, a thorough intra-articular knee examination is done to assess possible concomitant intra-articular injuries. Subsequently, the 30° arthroscope can be advanced in the posteromedial compartment through the Gillquist portal. The double posteromedial portal is established while examining the posterior compartment through the Gillquist portal. After, through both the Gillquist portal and posteromedial portal, the fracture is exposed by releasing some of the soft tissue remnants, fracture debris, and blood clots using an arthroscopic shaver and a rasp. Then, an arthroscopic posterior cruciate ligament tibial drill guide is passed through the anteromedial portal and intercruciate space to reduce the fracture while performing anterior tibial translation with the knee in 90° of flexion. The reduced fracture fragment is then pinned in position using the accompanying 2.4-mm guide pin under direct arthroscopic visualization via the posteromedial portal. This is then over-reamed with a 4.5-mm reamer. The reamer is then left in situ and the guide pin is removed. A wire loop is then introduced through the reamer into the posterior compartment and retrieved from the posteromedial portal. A suspensory device is then relayed through the tibia tunnel with a suture shuttle. The oblong button is then flipped onto the bony fragment under direct arthroscopic visualization. A button on the anteromedial tibia is then tensioned by sequentially pulling on both limbs of the device to fix the fracture in its reduced position. The knee is cycled between flexion and extension multiple times to assess the tension given through the suspensory device under direct arthroscopic visualization. The tensioning sutures are then tied with a Nice knot before completing multiple half hitches to secure the fixation.
